# Agro-ecosystems impact malaria prevalence: large-scale irrigation drives vector population in western Ethiopia

**DOI:** 10.1186/1475-2875-12-350

**Published:** 2013-10-02

**Authors:** Kassahun T Jaleta, Sharon R Hill, Emiru Seyoum, Meshesha Balkew, Teshome Gebre-Michael, Rickard Ignell, Habte Tekie

**Affiliations:** 1 Department of Zoological Sciences, Addis Ababa University, Addis Ababa, Ethiopia; 2 Department of Biological Sciences, Wollega University, Nekemte, Ethiopia; 3 Department of Plant Protection Biology, Unit of Chemical Ecology, Swedish University of Agricultural Sciences, Alnarp, Sweden; 4 Aklilu Lemma Institute of Pathobiology, Addis Ababa University, Addis Ababa, Ethiopia

**Keywords:** *Anopheles arabiensis*, *Plasmodium falciparum*, *Plasmodium vivax*, Malaria prevalence, Biting rate, Sporozoite rate, Entomological inoculation rate, Ethiopia

## Abstract

**Background:**

Development strategies in Ethiopia have largely focused on the expansion of irrigated agriculture in the last decade to reduce poverty and promote economic growth. However, such irrigation schemes can worsen the socio-economic state by aggravating the problem of mosquito-borne diseases. In this study, the effect of agro-ecosystem practices on malaria prevalence and the risk of malaria transmission by the primary vector mosquito, *Anopheles arabiensis*, in Ethiopia were investigated.

**Methods:**

In three villages in western Ethiopia practising large-scale sugarcane irrigation, traditional smallholder irrigation and non-irrigated farming, cross-sectional parasitological surveys were conducted during the short rains, after the long rains and during the dry season. Entomological surveys were undertaken monthly (February 2010-January 2011) in each village using light traps, pyrethrum spray collections and artificial pit shelters.

**Results:**

Malaria prevalence and the risk of transmission by *An. arabiensis* assessed by the average human biting rate, mean sporozoite rate and estimated annual entomological inoculation rate were significantly higher in the irrigated sugarcane agro-ecosystem compared to the traditionally irrigated and non-irrigated agro-ecosystems. The average human biting rate was significantly elevated by two-fold, while the mean sporozoite rate was 2.5-fold higher, and the annual entomological inoculation rate was 4.6 to 5.7-fold higher in the irrigated sugarcane compared to the traditional and non-irrigated agro-ecosystems. Active irrigation clearly affected malaria prevalence by increasing the abundance of host seeking *Anopheles* mosquitoes year-round and thus increasing the risk of infective bites. The year-round presence of sporozoite-infected vectors due to irrigation practices was found to strengthen the coupling between rainfall and risk of malaria transmission, both on- and off-season.

**Conclusion:**

This study demonstrates the negative impact of large-scale irrigation expansion on malaria transmission by increasing the abundance of mosquito vectors and indicates the need for effective vector monitoring and control strategies in the implementation of irrigation projects.

## Background

In Ethiopia, development strategies in the last decade have largely focused on the expansion of irrigated agriculture. The implementation of irrigation development schemes is one of the most effective ways to reduce poverty and promote economic growth [1]. These schemes raise crop production through enhanced yield, acreage and number of cropping cycles per year, as well as decrease the risk of crop failure [2]. Policy makers emphasize that the increased availability of irrigation and the lowered dependency on rain-fed agriculture is an effective means to increase food production and enhance the self-sufficiency of the rapidly increasing human population [3,4].

Despite these socio-economic benefits, irrigation development schemes can aggravate the problem of mosquito-borne diseases by increasing the number of aquatic larval habitats and extending the duration of the transmission season [5,6], or by removing the seasonal cycling all together [7]. A recent study has also indicated that the expansion of irrigation, and thereby agriculture, might result in the invasion and establishment of malaria vectors into previously vector-free areas [8]. The introduction of irrigation schemes, and associated agricultural practices, are now considered to be among the primary factors driving the increase in the global malaria burden [7]. The mechanisms underlying this phenomenon remain poorly understood given the complexity of vector ecology, parasite transmission and human behaviour.

Globally, mosquito populations have been shown to increase substantially following the introduction of large-scale irrigation (e g, [9]). In areas with unstable or seasonal malaria transmission, the transmission rate generally increases significantly following the introduction of large-scale irrigation [9-12]. In contrast, in areas with existing high endemic malaria transmission, the rates generally remain the same [13,14], or may even decrease following the implementation of large-scale irrigation [7,15-19].

In central Ethiopia, where malaria transmission intensities vary seasonally, malaria incidence and density of the primary malaria vector in the region, *Anopheles arabiensis*, is higher in villages associated with irrigated areas compared to non-irrigated areas [20,21]. Furthermore, in the highlands of Tigray, northern Ethiopia, where malaria transmission is seasonal, Ghebreyesus *et al.*[22] reported increased risk of malaria in newly developed irrigated areas following the construction of purpose-built small dams. Given the increased rate of expansion of irrigation schemes in most parts of Ethiopia, and the limited studies on the association between irrigation expansion and malaria transmission in the country, further investigations are required to evaluate the impact of irrigation on malaria prevalence. The results of such studies provide baseline data for the selection and implementation of appropriate malaria and vector control measures.

In this study, the effect of agro-ecosystem on malaria transmission and the abundance and epidemiological significance of the primary malaria vector, *An. arabiensis* were investigated in three villages practising large-scale sugarcane irrigation, traditional smallholder irrigation or non-irrigated agriculture in western Ethiopia.

## Methods

**Figure 1:**
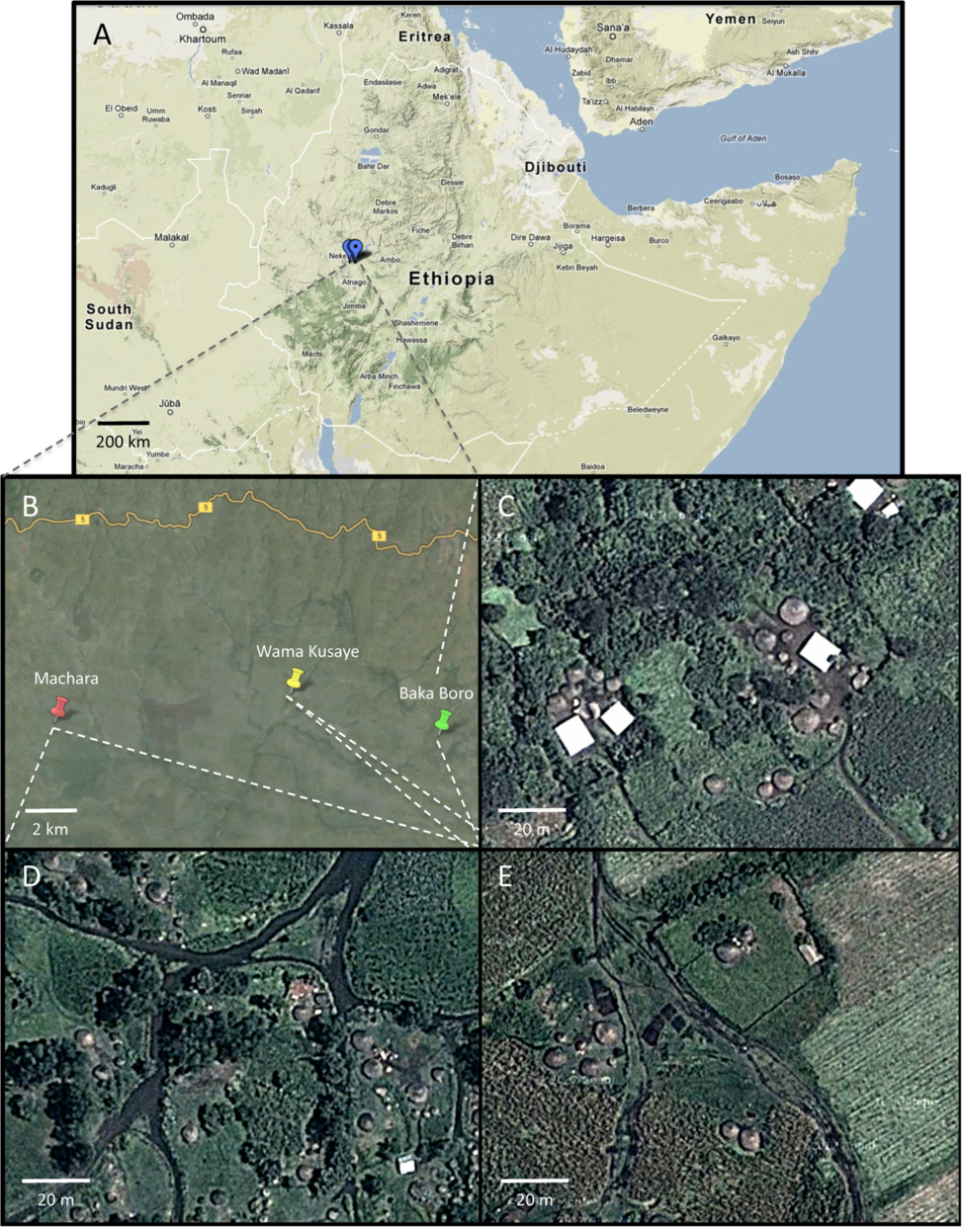
**Map of the study area.** Map of the location of the study sites in Ethiopia **(A)** in the East Wollaga district of Sibu Sire **(B)** consisting of three villages, Baka-Boro **(C)**, Machara **(D)** and Wama Kusaye **(E)**, which practice traditional irrigation, no irrigation and sugarcane plantation irrigation, respectively.

This study was conducted in three villages, located 8–17 km apart, in the Sibu Sire district in East Wollega Zone, western Ethiopia (Figure 1). The area has typically two rainy seasons: a long rainy season from June to September, with the peak rainfall in July and August, and a short rainy season from April to May. Of the population in this region, 87% (approximately 16,470 households with an average of six persons per household) are rural inhabitants, farming on average 1 ha of land per household. The vast majority of these households rely on subsistence agriculture for food and income (Ebba, 2008). The average yearly income from agricultural production in this region in 2005 was 5,120 Birr (ca. US$270) per household (Ebba, 2008). The three villages in this study are situated directly within the agro-ecological landscape and the vast majority (>90%) of the population of each village lives in households engaged in subsistence agriculture of mixed crop and livestock farming systems. The houses are closely surrounded by animal enclosures and agricultural fields (Figure 1C,D,E). Long lasting insecticide-treated nets (LLINs) are distributed periodically free of charge to households in the three villages and there are on average 2 LLINs per household (Sibu Sire Health Centre). As part of an anti-malaria campaign, every household is subjected to indoor residual spraying (IRS) once a year immediately after the peak of the long rains in July or August.

Wama Kusaye village (8°58.695’ N, 36°48.558’ E; 1,443 m above sea level) is situated within a large-scale irrigated sugarcane plantation of approximately 4,300 ha. The majority of the human habitation (population of 2,539 in 2010) is located in the centre of the plantation, with other houses surrounding the plantation in close proximity (Figure 1E). Most of the villagers (>90%) are farmers, mainly cultivating crops, including maize, sorghum and millet on a subsistence scale and keeping cattle (n = 2,903). These farmers use traditional irrigation methods for their own crops. Traditional irrigation in the Sibu Sire region consists of diverting existing rivers and tributaries into open gravity fed earthen ditches that extend around the houses and fields using locally accessible materials [23]. A minority of the villagers also work for the sugarcane plantation. Irrigation on the sugar plantation is accomplished by diverting Indris and Jalele rivers into open gravity fed concrete lined canals that ramify throughout the plantation. It is not uncommon for these canals to be filled with standing water or for leakage from the canals to occur. The canals and ditches also form potential mosquito breeding grounds during the long rains when they are not in use.

Baka-Boro (8°57.715’ N, 36°52.058’ E; 1,522 m above sea level; population of 1,810 in 2010) is a village practising traditional smallholder irrigation. Its agricultural fields and irrigation ditches are in close proximity to the houses (Figure 1C), and during the dry season, cabbage, tomato, onion, and potato are grown using traditional irrigation practices. During the rainy seasons, maize, sorghum, millet, and assorted vegetables are cultivated. The villagers keep cattle (n = 2,272) along with cultivating crops. Machara village (8°58.028’ N, 36°42.994’ E; 1,514 m above sea level; population of 2,349 in 2010) practices non-irrigation and rain-fed farming (Figure 1D), with maize, sorghum, millet and peppers grown at a subsistence level; and the villagers also keep cattle (n = 3,164) for their livelihood.

### Parasitological study

Cross-sectional parasitological surveys were conducted in the three study villages during the short rainy season (May 2010), after the long rainy season (October 2010) and during the dry season (February 2011) to determine the prevalence of malaria. In each village, thick and thin blood smears were taken from school-aged children aged from 4–15 years and living in the villages by mobilizing the parents through local administrative authorities to bring their children. Children were excluded from the study if they had taken anti-malarial medication within 4 days of testing or they did not have parental consent. Blood sample collection, preparation, staining, and microscopic identification of *Plasmodium* species were performed as previously described [24].

### Mosquito collection

Entomological surveys were conducted monthly in each village, from February 2010 to January 2011, using the Centers for Disease Control and Prevention (CDC) light traps (BioQuip Products, Inc, CA, USA), pyrethrum spray collections and artificial pit shelters. Indoor sampling of mosquitoes was performed in ten houses with similar sizes and construction patterns for each village. In ten houses in each village, a CDC light trap was hung beside an untreated bed net with an adult human volunteer sleeping under the bed net [25]. Only one occupant was in each house during each test. The light traps were set once every month and operated from 18:00 to 06:00 the following morning. In addition, sampling of indoor resting mosquitoes was conducted from 06:00 to 08:00 in another ten houses in each village, using pyrethrum spray collection [26]. Outdoor-resting mosquitoes were surveyed at five pit shelters (1.5x1.0x2.0 m) [26], one at the centre and the others equidistant around the periphery of each village. The artificial pit shelters were visited once a month for 15 min between 07:00 and 08:00 to collect resting mosquitoes using a mouth suction aspirator.

### Mosquito processing, identification and sporozoite detection

After collection, *Anopheles* mosquitoes were counted and then sorted by sex, abdominal condition (unfed, freshly fed, gravid and half gravid), and species using morphological keys [27]. Of the *Anopheles* mosquitoes that were provisionally identified as *Anopheles gambiae sensu lato*, 386 (>5%) were conclusively identified as *An. arabiensis* using the polymerase chain reaction (PCR) technique described by Scott *et al.*[28]. All *An. gambiae s. l.* caught in this study were then decidedly presumed to be *An. arabiensis*. This is consistent with previous investigation in Ethiopia [29-31] that showed there are no other *An. gambiae s. l.* malaria vectors in the region including the study area. In fact, the only other *An. gambiae s. l.* species to be found in Ethiopia is *An. amharicus*[32] (previously *An. quadriannulatus* species B [31]), which is restricted to altitudes above 1800 m in the Jimma district [31]. All unfed female *Anopheles* from the light trap collections were dissected under a dissecting microscope to determine the parous rate. The ovaries were examined and classified as parous or nulliparous based on the presence or absence of tracheolar skeins, respectively [33]. The head and thorax of parous *Anopheles* mosquitoes were stored individually in labelled vials with silica gel for further laboratory processing. To determine the sporozoite rate in host-seeking mosquitoes (parous from CDC traps), the head and thoraces were tested using the enzyme-linked immunosorbent assay (ELISA) technique for the presence of *Plasmodium falciparum* and *Plasmodium vivax* circumsporozoite proteins [34].

### Ethical considerations

Ethical clearance was obtained from the Ethical Committee of the Faculty of Science, Addis Ababa University conforming to the WMA Declaration of Helsinki. Informed consent was obtained from the parents of children involved in the study prior to taking blood samples. All malaria positive cases were treated according to the Ethiopian Ministry of Health Guidelines [35]. Verbal consent was obtained from both volunteers and household owners for light trap and pyrethrum spray mosquito collections.

### Data analysis

Malaria parasite prevalence was determined as the proportion of malaria positive cases expressed in percent. The average human biting rate was estimated as the number of mosquito bites/person/night from the CDC light trap catches using the formula by Lines *et al.*[36]. The parous rate was determined as the proportion of host-seeking mosquitoes (non-blood fed) caught in the CDC light traps that had ovaries with uncoiled tracheoles [33]. The sporozoite rates were calculated as the proportion of host-seeking mosquitoes that were ELISA positive [37]. The daily entomological inoculation rate (EIR) was determined by multiplying the mean number of mosquito bites/person/night by the proportion of those bites positive for sporozoites [37]. The annual EIR was then calculated by multiplying the mean number of human bites per night by the sporozoite rate and by 365 days. Chi square (*X*^2^) analyses were carried out to test for significance of malaria prevalence and mosquito catches among the study villages representing the different agro-ecosystems using SPSS® v. 16 (SPSS Inc., Chicago, IL, USA). Ordinal logistic regression analyses were carried out to test for significance of human biting rate, parous rate, sporozoite rates and EIR among the study villages representing the different agro-ecosystems using Minitab® v. 14 (Minitab Inc, State College, Pennsylvania, USA).

## Results

### Malaria prevalence

**Figure 2:**
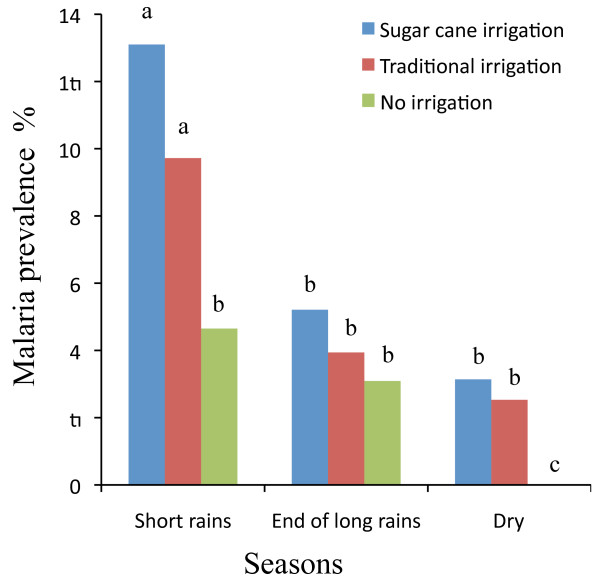
**Malaria prevalence in three agro-ecosystems practising large-scale irrigation, traditional irrigation and no irrigation, respectively.** Cross-sectional surveys were undertaken in the short rainy season (May 2010), after the long rainy season (October 2010), and during the dry season (February 2011). Different lower case letters indicate the significant difference (P <0.05).

In the parasitological surveys conducted during the short rainy season, after the long rainy season and during the dry season, a total of 879, 693 and 796 children were examined for malaria parasites in the sugarcane irrigated, the traditional irrigated and the non-irrigated agro-ecosystems, respectively. *Plasmodium falciparum* and *P. vivax* were the two parasite species detected during all parasitological surveys in all study villages. *Plasmodium falciparum* was the predominant malaria parasite (>65%), and *P. vivax* accounted for the remaining malaria prevalence (<35%) in all agro-ecosystems. In the two villages within the irrigated agro-ecosystems, malaria infections were recorded in all seasons (with annual 7.2 and 5.3%), with the highest prevalence occurring during the short rainy season, and the lowest at the end of the long rainy season and during the dry season (Figure 2). However, no significant differences (P >0.05) in malaria prevalence were observed between the two villages practising irrigation throughout the surveyed seasons (Figure 2). In the village within the rain-fed agro-ecosystem, annual malaria prevalence (2.9%) was significantly lower (P <0.05) than the two villages practising irrigation, annually as well as during the short rainy season and the dry season (Figure 2). In fact, during the dry season, no malaria (Figure 2) was observed in the village within the rain-fed agro-ecosystem. In contrast, malaria prevalence during the dry season was not significantly different from that observed after the long rainy season in the two villages within the irrigated agro-ecosystems (P >0.05; Figure 2).

### Species composition and abundance of malaria vectors

**Figure 3:**
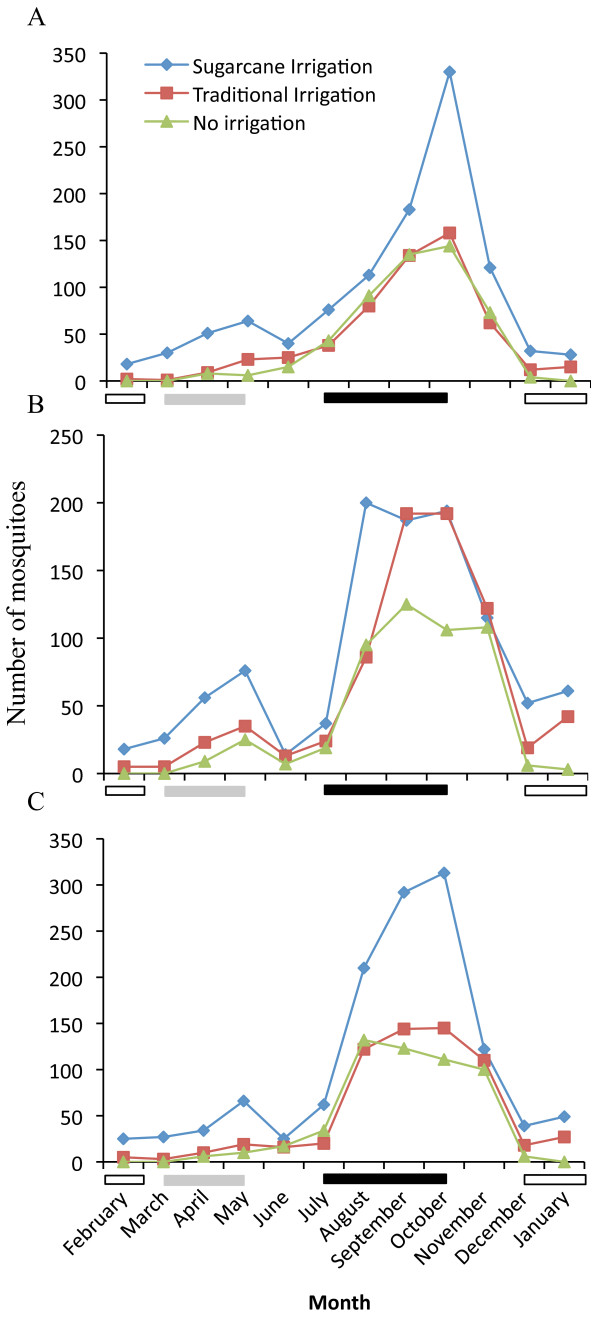
**Monthly caught *Anopheles arabiensis *in three agro-ecosystems practising large-scale irrigation, traditional irrigation and no irrigation, respectively.** Number of female mosquitoes caught by **(A)** CDC light traps, **(B)** pyrethrum spray sheet collections, and **(C)** artificial pit traps. The seasons are indicated with bars below the graphs: dry (white), short rains (gray) and long rains (black).

Four species of *Anopheles* mosquitoes including *An. arabiensis*, *Anopheles funestus s. l.*, *Anopheles nili*, and *Anopheles coustani*, were collected and identified in the study area (n = 7008). *Anopheles arabiensis* was the most abundant species comprising more than 98.5% of the specimens in the study area, and was the only member of the *An. gambiae s. l.* complex to be identified following PCR analyses of 386 female mosquitoes. A total of 6,903 female *An. arabiensis* were collected from the study villages during the monthly sampling, using CDC light traps (n = 2,164), pyrethrum spray sheet collections (n = 2,297) and artificial pit shelters (n = 2,442). The different collection methods revealed the presence of *An. arabiensis* throughout the year in the irrigated areas, whereas no mosquitoes were caught in the non-irrigated area during the peak of the dry season (Figure 3). The majority of mosquitoes caught by each of the three collection methods were from the latter half (August-September) to shortly after the end (October-November) of the long rainy season, in each of the agro-ecosystems (Figure 3). Monthly catches decreased thereafter until the onset of the short rainy season during which a small increase in the population was observed (Figure 3). About half of all the *An. arabiensis* mosquitoes (49.1%) were collected from the village in the irrigated sugarcane plantation, while the collections from the villages practising traditional irrigated or non-irrigated farming, totalled 28.3 and 22.6%, respectively.

The total number of host-seeking *An. arabiensis* caught by CDC light traps (Figure 3A) in the sugarcane irrigation agro-ecosystem was significantly higher than that found in the traditional irrigated (*X*^2^ = 9.916, df = 1; P < 0.005) and non-irrigated agro-ecosystems (*X*^2^ = 13.43, df = 1; P < 0.001). Furthermore, there was a significantly higher total number of indoor-resting *An. arabiensis* in the sugarcane-irrigated agro-ecosystem than in either the traditional (*X*^2^ = 24.37, df = 1; P <0.0001) or the non-irrigated agro-ecosystems (*X*^2^ = 12.55, df = 1; P < 0.001) (Figure 3B). Similar to that observed for host-seeking and indoor-resting *An. arabiensis*, the total number of outdoor-resting mosquitoes was significantly higher in the sugarcane irrigated agro-ecosystem compared to the traditional irrigated (*X*^2^ = 18.80, df = 1; P <0.0001) and the non-irrigated agro-ecosystems (*X*^2^ = 5.785, df = 1; P <0.05) (Figure 3C). No significant difference was observed in the total number *An. arabiensis* caught using all collection methods between the traditional and non-irrigated agro-ecosystems (P >0.05).

### Biting rate

**Figure 4:**
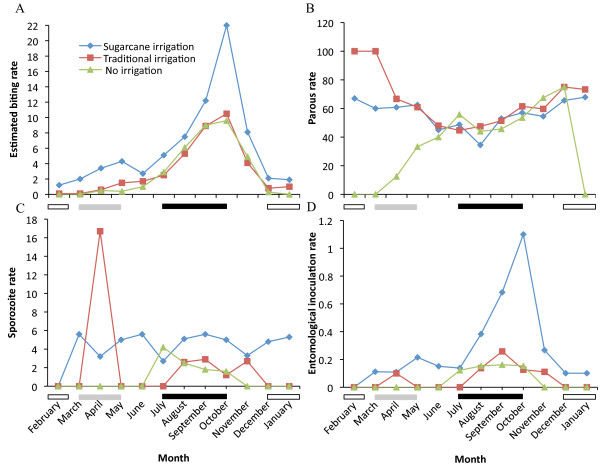
**Overall monthly entomological factors that affect the risk of malaria infection by *Anopheles arabiensis *in three agro-ecosystems practising large-scale irrigation, traditional irrigation and no irrigation, respectively. (A)** Estimated human biting rate, **(B)** parous rate, **(C)** sporozoite rate, and **(D)** entomological inoculation rate. The seasons are indicated with bars below the graphs: dry (white), short rains (gray) and long rains (black).

The mean monthly human-biting rate of *An. arabiensis* varied among the three agro-ecosystems (Figure 4A; *X*^2^ =8.912, df =2, P =0.012). The probability of being bitten by *An. arabiensis* in the irrigated sugarcane village was six times higher (95% CI = 1.18-31.09) than both the traditionally irrigated village (coefficient 1.801, z = 2.16; P =0.031) and 14 times higher (95% CI = 2.34-82.82) than the non-irrigated village (coefficient 2.634, z = 2.90; P =0.004). In all agro-ecosystems, the month of the year affects the biting rate (coefficient −0.424, z = −3.58; P <0.0001). The biting rate increased substantially from June to October, and then gradually decreased and reached minimal values during the dry season. Although it was of a lower magnitude, there was an increased human-biting rate during the short rains compared to the long rainy season in all the agro-ecosystems. The highest overall mean human-biting rate was recorded from the irrigated sugarcane agro-ecosystem (6.04 bites/person/night, 95% CI = 2.24-9.84) followed by the traditionally irrigated (3.09 bites/person/night, 95% CI = 0.88-5.30) and the non-irrigated agro-ecosystems (2.89 bites/person/night, 95% CI = 0.59-5.19).

### Parous rate

Non-blood fed host-seeking *An. arabiensis* females caught by CDC light traps in the sugarcane irrigated (n = 1,086), the traditional irrigated (n = 559), and the non-irrigated agro-ecosystems (n = 519) were dissected to determine their parous status. A parous rate of approximately 45% was observed during the long rainy season in the two irrigated agro-ecosystems (Figure 4B). During the dry and short rainy seasons, however, the parous rate increased in both of the irrigated agro-ecosystems (60-100%) (Figure 4B). The probability of a host-seeking female being parous did not differ between the irrigated sites (coefficient-0.408, z = −0.50; P =0.621) however the there was a six times lower probability of a host-seeking female being parous in the non-irrigated village (coefficient 1.808, z = 2.15; P =0.031). The parous rate in the non-irrigated agro-ecosystem gradually increased from nil at the onset of the short rainy season to approximately 75%, three months after the end of the long rainy season (Figure 4B). Thereafter, the parous rate decreased substantially. While the probability of a host-seeking female being parous on any of the three sites was not affected by the month of the year (coefficient −0.143, z = −1.47; P =0.142), the extreme differences in parous rates during the dry season in the non-irrigated village (Figure 4B) is a result that during these 3 months there were no mosquitoes (thus the parous rate is 0). Also, there were very few mosquitoes in February (n = 2) and in March (n = 1) in the traditionally irrigated village, all of which are parous (therefore 100% parous rate). This suggests that the only region in which the mosquito populations are being locally replenished during this period is the sugarcane irrigation village. Following the onset of the short rains and through to the end of the long rains, the parous rate, and thus presumably the rate of taking vertebrate blood meals, does not differ among the three villages.

### Malaria sporozoite and entomological inoculation rates

The monthly sporozoite rates for *An. arabiensis* varied among all three agro-ecosystems (Figure 4C). In the irrigated sugarcane agro-ecosystem, sporozoite-positive *An. arabiensis* were recorded throughout the year, except in February. In the traditionally irrigated agro-ecosystem, a very high sporozoite rate (16.7%) was observed in April, with lower rates (ca. 1-3%) recorded at and after the end of the long rainy season, between August and November. In contrast, in the non-irrigated agro-ecosystem, sporozoite-positive mosquitoes were only recorded during, and immediately after, the long rainy season, from July to October. Significant differences in the overall sporozoite rate for *An. arabiensis* were observed among the three agro-ecosystems (*X*^2^ =13.104, df =2; P =0.001); an annual rate approximately 2.5-fold higher was observed in the irrigated sugarcane agro-ecosystem (4.6%, n = 526) compared to the traditionally irrigated (2%, n = 300; coefficient = 2.629, z = 2.74; P =0.006) and the non-irrigated (1.7%, n = 237; coefficient = 4.409, z = 3.37; P =0.001) agro-ecosystems.

The risk of a person receiving infective bites from sporozoite-infected female *An. arabiensis* was 4.6-fold higher in the irrigated sugarcane agro-ecosystem, with an estimated annual EIR of 102 infective bites/ person/year, than in the traditional irrigated agro-ecosystem (22 infective bites per year; coefficient =3.689, z =3.33; P =0.001) and 5.7-fold higher than the estimated 18 infective bites per year in the non-irrigated agro-ecosystem (coefficient =3.906, z =3.42; P =0.001). The probability of receiving an infective bite was affect by the time of year (coefficient −0.397, z = −3.06; P =0.002) with the highest risk of an infective bite occurred between July and December in all three agro-ecosystems (Figure 4D).

## Discussion

The increasing implementation of irrigation in response to governmental policies causes considerable change in agro-ecosystems, which raises important questions concerning the accompanying changes in malaria transmission dynamics and its association with agricultural practices. In this study, malaria prevalence, vector abundance, and the factors affecting the EIR were investigated in villages situated within agro-ecosystems employing different irrigation schemes. Previous studies have shown variability in malaria prevalence following the implementation of active irrigation schemes. In some cases, malaria prevalence increases [9,10,12,21,22,38-44], while in others it can decrease or even remain unchanged [7,13-19,45,46]. Such variation suggests that the effects of irrigation on the factors underlying malaria transmission are poorly understood. In the present study, active irrigation clearly affected malaria prevalence by increasing the abundance of sporozoite-infected host seeking mosquitoes year-round, thus increasing the risk of receiving infective bites. The year-round presence of sporozoite-infected vectors due to irrigation practices was found to strengthen the coupling between rainfall and risk of malaria outbreak, both on- and off-season.

Previously, researchers have outlined a simple overall model for the increased malaria prevalence in irrigated areas [9,10,12,21,22,38-44]. Irrigation increases the number of suitable breeding sites persisting throughout the year, leading to increased and persistent vector abundance. Higher vector population densities mean increased human biting rates, parous rates and sporozoite infections, resulting in increased EIRs, and, in regions without ongoing vector protection, increased malaria prevalence. The main findings from this study support this model. Throughout the study period in the study sites, the predominant malaria vector was from the *An. gamibiae s. l.* and was determined to be *An. arabiensis* using PCR verification and supporting the findings that only *An. arabiensis* are found in this region of Ethiopia [29-31]. *Anopheles arabiensis* females were collected all year round in both traditional and sugarcane irrigated villages indicating the availability of mosquito breeding habitats all year round in villages practising irrigation. However, *An. arabiensis* females were not found in traps in the non-irrigation agro-ecosystem during the dry season, presumably due to the unavailability of suitable breeding habitats in and around the village. This indicates that irrigation has weakened, if not removed, the seasonal cycling of malaria vector abundance observed in the non-irrigated village, similar to what has been previously shown [12,21].

Even though *An. arabiensis* was found throughout the year in both of the irrigated villages, abundance and biting rate in sugarcane irrigated agro-ecosystem was significantly higher than that of the traditional irrigation agro-ecosystem. The sporozoite rate and resulting EIR of *An. arabiensis* in the three agro-ecosystems showed variations similar to mosquito abundance and biting rates, with higher sporozoite rate observed in sugarcane irrigation than the traditional irrigation and non-irrigated agro-ecosystems. These findings suggest a higher risk of malaria transmission in the sugarcane irrigated agro-ecosystem. In line with these studies, Githeko *et al.*[19] and Ijumba *et al.*[17] have reported high sporozoite rates and EIRs in villages associated with surface-irrigated sugarcane plantations in Miwani, Kenya and Mvuleni, Tanzania.

The findings in the parasitological surveys of this study are in agreement with the entomological data in that there was a strong positive correlation between malaria prevalence and sugarcane irrigation. Several African countries cultivate sugarcane for production of sugar or fuel alcohol [6]. Many action plans for increased sugarcane production and improvement over the next decade are currently in place in these countries, and the majority of these initiatives name the development of irrigation infrastructure as an essential component to their success. There are, however, only a few studies on the impact of sugarcane irrigation practices on malaria transmission in Africa (e g, [17,19]). That said, without accurate monitoring and vector control initiatives in place, there is the risk of a resurgence of malaria transmission as a result of sugarcane irrigation development, such as has been reported previously [47-49].

Irrigation, both traditional and large scale, resulted in significantly higher annual malaria prevalence than observed in the solely rain-fed agro-ecosystem. The agro-ecosystems under discussion contain villages of 1,500 to 2,500 inhabitants with many cultivations within the borders of the villages, as well as being surrounded by a larger agricultural landscape. Several investigations have found that malaria prevalence is higher in such agro-ecosystems when traditional and/or more large-scale irrigation techniques are employed [12,21,38]. This higher annual malaria prevalence in irrigated areas can be attributed to differences during two of the three seasons. First, malaria prevalence is higher during, and in some cases throughout, the dry season in irrigated areas [21] whereas it is lower or absent in the rain-fed regions [12,21, this study]. For example, in western [this study] and central Ethiopia [21], remarkably higher malaria prevalence was found to extend into the dry season in villages practising irrigation compared to villages without irrigation. Second, during the short rains there is a substantially higher malaria prevalence in villages using irrigation, compared to those which are not [this study]. In this study, the malaria prevalence more than doubled during the short rains in irrigated compared to rain-fed agro-ecosystems. The persistence of increased levels of malaria during the dry season and the short rains means that in the villages with irrigation schemes in practice, the burden of malaria is maintained throughout the year. This is in stark contrast to the non-irrigated villages in which the burden is substantially lower during the dry (this study, [12,21]) and short rainy seasons (this study, [12]). This shift from season-based to year-round malaria transmission in newly developed irrigated areas has been reported in Ethiopia (this study, [21]–[22]) and throughout much of the tropics and sub-tropics [9,10,12,38-44].

Contrary to the findings of this study, there are a few studies demonstrating that irrigation was not associated with higher malaria prevalence [13-19,45,46]. As most of the described cases were associated with irrigated rice fields [13-19,45], this phenomenon has been called the “paddies paradox” e g, [14], in which the mosquito abundance increased between four- and 300-fold, while the malaria prevalence was equal to, or lower than, neighbouring non-irrigated regions. A plausible explanation for this paradox is the stronger economic base available to those living in large-scale irrigation areas leading to inhabitants having an increased access to vector protection, e g, bed nets and indoor residual spraying (IRS) [14,17,45]. Investigations into the human biting rates in these regions support this hypothesis, as these rates are typically lower than observed for those of nearby non-irrigated villages [14,17,45]. In one instance, this appears to have resulted in a shift in the local host preference of the vectors from anthropophilic to zoophilic, where *An. arabiensis* local to irrigated rice fields in Kenya overwhelmingly preferred cattle over humans as hosts [14].

Studies that have demonstrated a negative correlation between malaria prevalence and sugarcane projects [17,46], may also be regulated by other underlying factors [46-50]. Even though sugarcane plantation needs irrigation for optimal growth, it does not require flooding as it is easily susceptible to water-logging [50]. Moreover, the thick vegetation cover resulting from sugarcane growth would make the environment unfavourable, as *An. gambiae* complex species prefer sunlight breeding habitats [6]. However, improperly maintained sugarcane irrigation water canals may create suitable breeding habitats for malaria mosquitoes. Ijumba and Lindsay [6] and Packard [49] also reported that lack of proper maintenance of the irrigation canals in sugarcane plantation may lead to the formation of suitable breeding sites for malaria vectors. The open water irrigation canals in sugarcane plantation of this study permit water leakage and are also the source of standing water that can favour the breeding of *An. arabiensis* mosquitoes throughout the year. The parous rates (ca. 60%) among the mosquitoes present during the dry season in the plantation-irrigated village indicate mosquitoes are continuing to breed in this area during the dry season. When compared to the absence of mosquitoes in the non-irrigated village and the 100% parous rate among the few aging mosquitoes found in the traditionally irrigated village, it is strongly indicated that the continuing population of mosquitoes in the plantation-irrigated village is a result of the presence of breeding sites associated with its irrigation practice. The consequences of maintaining a mosquito population through the dry season and providing good breeding sites in the disused irrigation canals during the long rains can be seen in the risk of receiving infective bites, which increases earlier in the year and peaks at a 4 to 6 times higher risk in the village surrounded by a plantation compared to the other villages. Such conditions, in turn, may contribute to the worsening of the malaria situation in the area. This strongly indicates that for effective malaria management in areas with large-scale irrigation projects, it is important to effectively manage the irrigation water year round.

Irrigation schemes, particularly large-scale ones such as used on sugarcane and rice plantations, strengthen the existing connection between rainfall and the risk of a malaria outbreak [7,18, this study]. In arid regions of north-western India it was found that irrigation on the large scale leads to more endemic conditions for malaria, which in turn creates the potential for unpredicted large epidemics following excessive rainfall, if vector control methods should coincidentally be relaxed [7]. The data from this study shows that the presence of an increased proportion of parous females during the dry season in irrigated areas was followed by a spike in the number and proportion of sporozoite-infected females during the short rains. This culminated in a significant increase in malaria infections at the end of the short rains in irrigated compared to rain-fed areas. It is important to note that during the short rains in this region of Ethiopia, the IRS programme is not yet underway. Therefore, the populace is less protected against this increase in the number of infective bites than during the long rains, when IRS is common. The risk of an infective bite during, and just after, the long rains is substantially higher in the large-scale irrigation region, where the sporozoite rate has remained high throughout the year. The spike in abundance of infected host-seeking mosquitoes during this time indicates that the intense risk of human malaria infection during this season is held in check by active vector control methods. Should these methods weaken or fail, the probability of a malaria outbreak is high. This increased necessity for reliance on vector control is evident as seen in the increased malaria prevalence in irrigated regions during the dry and short rainy season when vector control methods are relaxed.

In conclusion, this study indicated that malaria prevalence was higher and transmission continues throughout the year in irrigated villages than in non-irrigated villages in the study area. The parasitological findings are in line with the entomological data as it relates to malaria transmission parameters in the study area. The higher malaria prevalence and transmission in irrigated villages was likely the result of poor irrigation water management that led to availability of breeding sites all year round. This calls for proper management of water systems in irrigation projects year round. Therefore, policy makers should be aware of the potential risk of irrigation expansion in aggravating or causing the malaria transmission to become perennial. Moreover, when planning irrigation development schemes, decision makers should also plan appropriate options for the control of vector mosquitoes, to improve or establish health services and educate the local communities about personal protection measures and environmental management to avoid risk of mosquito-borne diseases including malaria.

## Abbreviations

CDC: Centers for disease control and prevention; ELISA: Enzyme-linked immunosorbent assay; EIR: Entomological inoculation rate; PCR: Polymerase chain reaction; X2: Chi square; s. l.: *Senso lato*; df: Degrees of freedom; CI: Confidence interval; IRS: Indoor residual spraying; LLINs: Long lasting insecticide-treated nets.

## Competing interests

The authors declare that they have no competing interests.

## Authors’ contributions

HT, ES and KTJ designed the study. HT supervised and KTJ conducted the fieldwork. MB and TG-M supervised and KTJ conducted the laboratory work. SRH conducted the logistic regression analyses, while KTH conducted the Chi square analyses. KTJ drafted the paper. SRH critically revised the manuscript. RI and HT critically reviewed the manuscript. All authors read and approved the final manuscript. HT and RI share final authorship responsibility. KTJ and SRH share first authorship responsibility.

## Authors’ information

Co-first authors: Kassahun Tadesse Jaleta and Sharon Rose Hill.

Co-last authors: Rickard Ignell and Habte Tekie.
